# Functional outcome in low-ASPECTS (0–5) acute ischemic stroke treated with mechanical thrombectomy: impact of laterality explored in a single-center study

**DOI:** 10.3389/fneur.2023.1205256

**Published:** 2023-07-04

**Authors:** Victor Dumas, Killian Martin, Clément Giraud, Julia Prigent, William Bloch, Karim Soualmi, Guillaume Herpe, Samy Boucebci, Jean Philippe Neau, Rémy Guillevin, Stéphane Velasco

**Affiliations:** ^1^LabCom I3M, DACTIM-MIS Team, LMA CNRS 7348, Poitiers University Medical Center, Poitiers, France; ^2^Department of Radiology, Poitiers University Medical Center, Poitiers, France; ^3^Department of Neurology, Poitiers University Medical Center, Poitiers, France

**Keywords:** stroke, mechanical thrombectomy, low-ASPECT, AIS-LVO, laterality

## Abstract

**Background:**

There is no consensus regarding the influence of infarct laterality in patients with acute ischemic stroke due to anterior large vessel occlusion (AIS-LVO) treated with mechanical thrombectomy (MT), particularly in low-ASPECT (0–5) patients who were excluded from the initial MT studies and that participated in dedicated randomized-controlled trials that do not consider the side of the occlusion. We aimed to evaluate the role of infarct laterality on the clinical outcome in low-ASPECT AIS patients treated with MT.

**Material and methods:**

We retrospectively analyzed our institutional stroke database in our Thrombectomy-Capable Stroke Center (TCSC), including patient characteristics, procedural variables, and outcomes, between January 2015 and January 2022. Patients with acute intracranial ICA and/or proximal MCA occlusions with ASPECT ≤ 5 either on CT or MRI were included and divided into 2 groups according to the location of ischemia. The primary endpoint was a good clinical outcome at 90 days (modified Rankin Scale (mRS) score of 0–3).

**Results:**

Between January 2015 and November 2021, 817 MT were performed, of which 82 were low-ASPECT (10.0%): 41 left-sided and 41 right-sided strokes. The rates of good clinical outcome were 30.8% (12/41) for the left-sided group and 43.6% (17/41) for the right-sided group, with a *p*-value of 0.349. The morality rate showed no significant difference between the two groups: 39.0% (16/41) in the right stroke group and 36.6% (15/41) in the left stroke group.

**Conclusion:**

The clinical outcome was not significantly influenced by stroke laterality. The results of this single-center retrospective study indicate either a lack of strength or equal value in performing mechanical thrombectomy regardless of stroke laterality.

## 1. Introduction

Acute ischemic stroke due to large vessel occlusion (AIS-LVO), whose therapeutic strategy has recently been revolutionized by the emergence of mechanical thrombectomy (MT), is one of the leading causes of death and disability worldwide (1, 2). Large-volume AIS-LVO represents a significant proportion of all strokes and is correlated with higher mortality and higher post-stroke disabilities. ASPECTS is the main score used both in computed tomography (CT) and magnetic resonance imaging (MRI) to evaluate the extent of AIS-LVO, and large core volume strokes are commonly considered with ASPECTS ranging from 0 to 5 (3). Since few of these patients were enrolled in the first randomized controlled clinical trials (RCTs), the benefit of MT in this setting was uncertain until recently (4–10); however, three recent RCTs have demonstrated the positive outcomes of MT in this context, and additional trial results are expected in the near future, placing these patients at the forefront of current challenges in stroke management (11–14). In this context, the influence of stroke laterality, which was historically a major and early subject of stroke studies and much studied in the post-thrombolysis era, is unclear (15–17). This study aimed to evaluate the impact of infarct laterality in patients with low-ASPECTS AIS-LVO who underwent MT.

## 2. Materials and methods

### 2.1. Patient selection and characteristics

**Figure 1:**
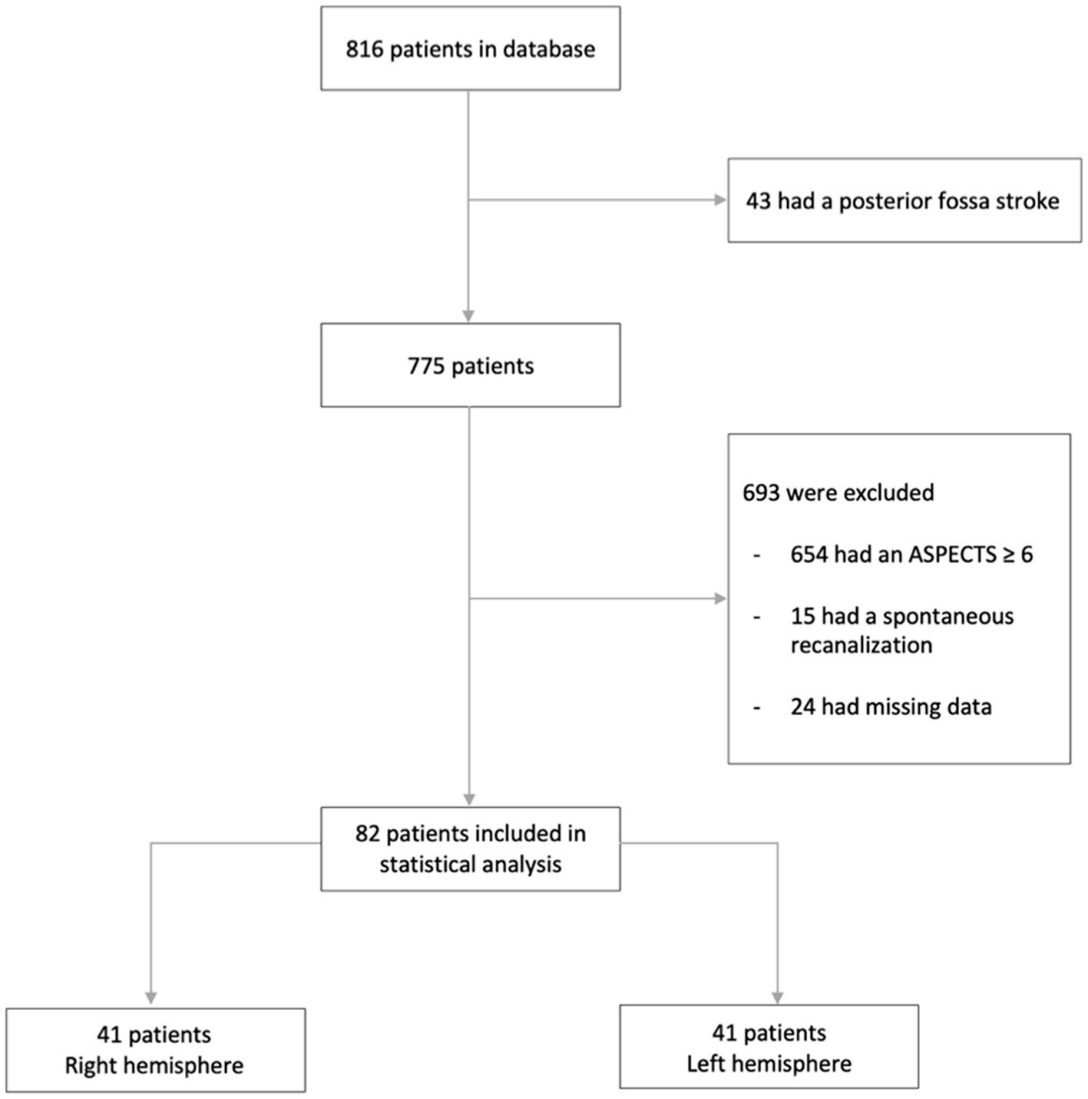
Flow chart.

We retrospectively analyzed all consecutive patients with AIS-LVO who underwent MT at a single Thrombectomy-Capable Stroke Center (TCSC) from January 2015 to November 2021. The inclusion criteria were acute occlusion of the intracranial ICA and/or proximal middle cerebral artery, including a proximal branch of the M2 segment on CT or MRI, treatment with thrombectomy, and an ASPECT score of ≤ 5 either on CT or MRI. Individuals younger than 18 years, patients with posterior circulation strokes, or those who experienced spontaneous recanalization were not included in the analysis (Figure 1).

### 2.2. Clinical and radiological data

For each patient, the recorded data included age, gender, medical history, baseline NIHSS, presence of major deficits such as aphasia and neglect, pre-stroke mRS, antiplatelet and anticoagulant treatments, stroke etiology, and symptomatic bleeding events (defined by a neurologic worsening, an increase in an NIHSS score of 4 or more, and evidence of intracranial hemorrhage on imaging). NIHSS and pre-stroke mRS were conducted by neurologists at the stroke center. The clinical outcome was based on the 90-day follow-up period, which could take place either at the same hospital where the mechanical thrombectomy was performed or at the center to which the patient was transferred. The neurologists conducted the assessment through physical examination or a telephone conversation. Technical success was evaluated by the radiologist who conducted the procedure and documented the assessment in the MT report. Radiological characteristics, including diagnostic and angiographic imaging, were retrieved from the local PACS and analyzed. Concerning radiological imaging, we assessed modality, thrombus location, supra-aortic trunk imaging, ASPECT, and stroke volume. The 10 brain regions (caudate, lentiform nucleus, internal capsule, insula, and six cortical regions) used to calculate the ASPECT score were inspected using the image set from each study. Out of a possible total score of ten, one point was deducted for each affected region. The images were evaluated independently by a senior neuroradiologist and a radiology resident. In cases of disagreement, a consensus was reached with the help of Rapid AI software. Stroke volumes were determined using Rapid AI software in the case of MRI and with semi-automatic contouring via Vitrea software in the case of CT scans.

### 2.3. Definition of outcomes

Successful reperfusion was defined as achieving a modified thrombolysis in cerebral infarction (mTICI) score of ≥ 2b. A good clinical outcome was determined by a modified Rankin Scale (mRS) score ranging from 0 to 3.

### 2.4. Study endpoint

Good clinical outcome according to stroke laterality was the study endpoint.

### 2.5. Statistical analysis

The characteristics of the population were obtained using different tests: the Student *t*-test or the Wilcoxon–Mann–Whitney test for continuous variables and the chi-squared and Fisher's exact test for categorical variables. Continuous variables were presented as the mean +/– standard deviation (SD). Categorical variables were presented as numbers (the corresponding percentage). These variables allowed us to determine whether patients had similar characteristics in the two groups. For all analyses, we considered the commonly used threshold of 005 for the type 1 error risk. The effect of stroke laterality and affected hemisphere on radiological, clinical, and hemorrhagic risks was studied using the chi-squared test. We compared the modified Rankin Score at 90 days in the right and left stroke groups using the chi-squared test. Then, adjustments were made with quantile regression models. The adjustment factors were the stroke volume and the major symptoms, i.e., the presence of aphasia and neglect at the onset.

## 3. Results

### 3.1. Study sample

Between January 2015 and January 2022, 817 MT were performed, of which 82 were low-ASPECT (10.0%): 41 left-sided strokes (50.0%) and 41 right-sided strokes (50.0%) (Table 1). The mean age of study subjects was 69 years old (±SD 15). A total of 46 patients were women (34.1%). In our sample, 96.7% of the patients were right-handed, with two left-handed people in the left stroke group and no left-handed people in the right stroke group. The groups were comparable regarding history, including high blood pressure, diabetes, and atrial fibrillation. We also determined that seven patients in each group had a history of stroke. The NIHSS score was higher in the left group without being significantly different (19 vs. 18 on the right, *p* = 0.064). The median time from symptom onset to reperfusion was 375 min in the left stroke group and 373 min in the right stroke group (*p* = 0.178). The median ASPECT score was 5, and the mean volume was 96.4mL; neither were significantly different between groups.

**Table 1:** Demographic and clinical characteristics of the patients at baseline.

**Laterality**	**Left (*N* = 41)**	**Right (*N* = 41)**	**Overall**	**Unknown**	***P* value**
**Age—** ***yr***	68 ± 17	70 ± 14	69 ± 15	0	0.438
**Hand—** ***no (%)***					
Left Handed	2 (6.5)	0 (0)	2 (3.3)	25.6	0.492
Right Handed	29 (93.6)	30 (100)	59 (96.7)	25.6	
**Male Sex—** ***no (%)***	28 (68.3)	26 (63.4)	54 (65.9)	0	0.816
**History—** ***no (%)***					
Hypertension	24 (58.5)	22 (53.7)	46 (56.1)	0	0.824
Diabetes	6 (14.6)	4 (9.8)	10 (12.2)	0	0.736
Active smoking	15 (36.6)	7 (17.1)	22 (26.9)	0	0.081
Hyperlipidaemia	10 (24.4)	11 (26.8)	21 (25.6)	0	1
Previous ischemic stroke	7 (17.1)	7 (17.1)	14 (17.1)	0	1
BMI	26.3 ± 4.9	26.0 ± 5.0	26.2 ± 4.9	2.4	0.784
Obesity (BMI > 29.9)	6 (14.6)	8 (20.5)	14 (17.5)	2.4	0.691
Atrial fibrillation	17 (41.5)	17 (41.5)	34 (41.5)	0	1
Chronic renal failure	4 (9.8)	5 (12.2)	9 (11.0)	0	1
Myocardial infarction	9 (22.0)	7 (17.1)	16 (19.5)	0	0.781
**Pre-stroke mRS (IQR)**	0 (0–0)	0 (0–0)	0 (0–0)	0	0.641
**NIHSS**	19 (13–25)	18 (14–22)	18 (13–23)	8.5	0.064
**Interval between time of stroke onset and time of reperfusion**					
Median (IQR)—min	375 (245–505)	373 (253–493)	374 (249–499)	4.9	0.178
<6.0 hr	17 (41.5)	12 (32.4)	29 (37.2)		0.488
6.0 to 24 hr	24 (58.5)	25 (67.6)	49 (62.8)		0.488
**Occlusion site—** ***no (%)***				0	
M1	24 (58.5)	26 (63.4)	46 (61.0)		
M2	3 (7.3)	5 (12.2)	8 (9.8)		
ICA terminus	12 (29.3)	7 (17.1)	19 (23.2)		
Tandem	2 (4.9)	2 (4.9)	4 (4.9)		
Extracranial ICA	0 (0)	1 (2.4)	1 (1.2)		
**ASPECT**					
Median value (IQR)	5 (5–3)	5 (5–4)	5 (5–4)	0	0.341
0—no (%)	1	0	1		
1—no (%)	1	0	1		
2—no (%)	3	4	7		
3—no (%)	7	4	11		
4—no (%)	7	9	16		
5—no (%)	22	24	46		
**Volume (mL)**	95.1 (77.4–120.2)	97.7 (78.1–121.0)	96.4 (78.9–121.8)	0	0.490
**General anesthesia—** ***no (%)***	16 (39.0)	15 (36.6)	31 (37.8)	0	1
**Intravenous rt-PA use—** ***no (%)***	23 (56.1)	26 (63.4)	49 (59.8)	0	0.652

### 3.2. Procedural metrics

The median time of onset-recanalization was 373 min (IQR 253–493) in the right-sided group and 375 min (IQR 245-505) in the left-sided group. Intravenous thrombolysis was administered to more than half of the patients in each group: 26 patients (56.1%) in the left stroke group and 23 patients (63.4%) in the right stroke group. MT was performed under general anesthesia for 15 patients in the right stroke group (36.6%) and 16 patients in the left stroke group (39.0%).

### 3.3. Outcomes

A total of 71 patients (86.6%) had an mTICI reperfusion score of 2b or higher with no significant difference between groups: 36 out of 41 (87.8%) for the left location and 35 out of 41 (85.4%) for the right location (*p* = 1) (Table 2). There was less than 25% hemorrhagic transformation in each group (nine patients in the left stroke group and seven patients in the right stroke group).

**Table 2:** Outcomes.

	**Laterality**		
	**Left stroke (41)**	**Right stroke (41)**	***P* value**
**Radiological**			
mTICI reperfusion grade ≥ 2b—*no (%)*	36 (87.8)	35 (85.4)	1
**Clinical**			
mRs *médian* at 3 months *(IQR)*	4 (3–6)	4 (2–6)	0.291
mRS median *(IQR)* adjusted with infarct size (mL)	4 (3–6)	4 (2–6)	0.958
mRS median *(IQR)* adjusted with the presence of neglect at J0	5 (4–6)	3 (2–6)	0.282
mRS median *(IQR)* adjusted if aphasia is present at J0	4 (3–6)	6 (6–6)	0.092
mRS median *(IQR)* adjusted with full adjustment	3 (2–6)	5 (4–6)	0.151
mRS ≤ 3 at 3 months—no (%)	12 (30.8)	17 (43.6)	0.349
Mortality at 3 months—*no (%)*	15 (36.6)	16 (39.0)	1
**Symptomatic hemorrhagic transformation—** ***no (%)***	9 (22.0)	7 (17.1)	0.781

### 3.4. Study endpoint

**Figure 2:**
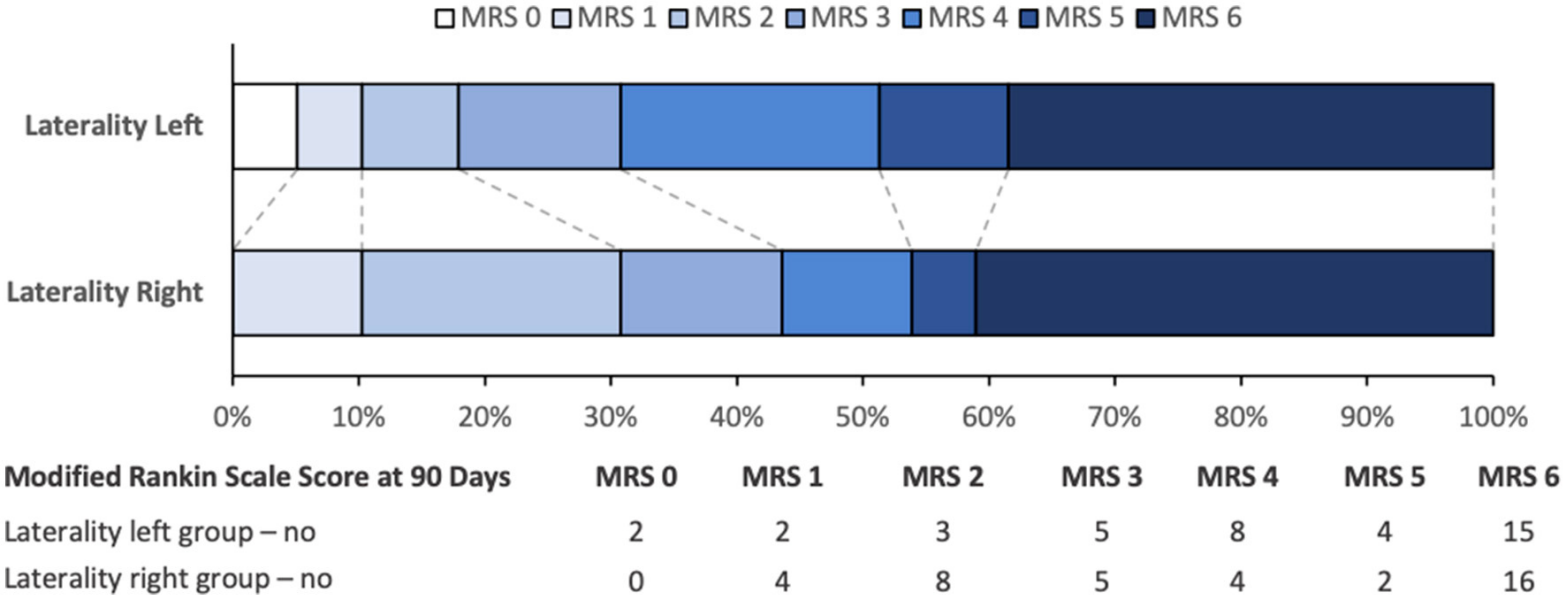
Modified Rankin scale scores at 90 days according to stroke laterality, A modified Rankin sclare score of 0 indicates no disability, 1 no clinically significant disability, 2 slight disability, 3 moderate disability but able to walk unassisted, 4 moderately severe disability, 5 severe disability, and 6 death.

The number of patients with a score of 0 to 3 on the modified Rankin scale at 90 days was 12 (30.8 %) in the left stroke group and 17 (43.6%) in the right stroke group, *p* = 0.349 (Figure 2). There were 15 deaths (36.6%) at 3 months in the left stroke group and 16 deaths (39.0%) in the right stroke group (*p* = 1).

## 4. Discussion

In this series, the percentage of patients with good functional outcomes at 90 days was not significantly different between patients with right and left hemispheric strokes. Nevertheless, a visual analysis of the arrangement of mRS scores 2, 3, and 4 at 3 months indicates that the prognosis may be more favorable for people with right-sided strokes. The lack of statistical significance does not allow us to draw any definitive conclusions, and further studies are needed to ascertain whether this effect exists, as we assumed before conducting this study. In particular, we believe that conducting a meta-analysis of patients included in previously published randomized trials (11–14) as well as upcoming trials is essential (TESLA, LASTE, and TENSION) to provide the necessary statistical power and the level of evidence needed to definitively address this question.

In our study, we chose a threshold of a good clinical outcome with an mRS score of 0 to 3. It is worth noting that the more traditional threshold for good clinical outcomes is an mRS score of 0 to 2. However, we chose the broader threshold of 0 to 3 to align with the focus on severe strokes, which is consistent with recently conducted RCT studies on low-ASPECT AIS-LVO patients (11–13). The use of the mRS in our study could have been problematic, as patients with low ASPECT scores are more likely to exhibit significant cortical region damage. Indeed, this score can be used to assess limitations in autonomy and daily activities, but it lacks specificity, and domains such as cognition, language, visual function, emotional disturbance, and pain are not directly measured. However, we believed that it was essential to use it as the primary endpoint for three main reasons: (1) The mRS is extensively used in the post-MT era, including among patients with low-ASPECT AIS-LVO (11–13), which strengthens the intrinsic and extrinsic validity of the study; (2) it has previously been shown to have correct concurrent validity with regard to infarct volumes, and its construct validity has been shown to have excellent concordance with other rating scales (18, 19); and (3) mRS endpoints generally require significantly smaller sample sizes to achieve adequate statistical power, and the odds of obtaining a statistically significant result increase by 89% with an mRS endpoint compared with a Barthel index endpoint (18).

The absence of any significant difference in clinical outcome at 3 months is consistent with subgroup analyses performed in a previously published meta-analysis (20) but is at odds with some others that described a better clinical outcome in patients with left-hemisphere stroke (16, 17). This association has been attributed to a larger infarct volume in right-vs.-left-hemisphere stroke patients (21) and a long time from the onset of stroke to its manifestation (22). Several studies have indeed highlighted that neglect increases the delay in stroke recognition and management (17, 23), but in our database, there was no longer a delay for right hemispheric stroke patients. This could be explained by the fact that low-ASPECT patients generally present a massive deficit that cannot be neglected.

On a similar note, neglect is often a determining marker that negatively influences functional recovery (24, 25), but this was not a finding in the mRS at 3 months in our sample.

Left-stroke patients in our trial had a non-statistically significant tendency to have a higher NIHSS score at admission, a notion that is concordant with other studies (21, 26). Thus, left hemispheric damage is often accompanied by language disorders that raise the NIHSS score compared with right hemispheric damage, associated with neglect, which is less weighted for the NIHSS score (27).

There were the same number of patients in each group. Thus, the laterality of stroke was not a factor influencing thrombectomy selection. We also observed no difference in the reperfusion rate between left and right strokes, thus laterality was not a factor in the failure of MT.

We failed to dichotomize our study population as having a major or minor hemisphere involvement because our sample included only two left-handers, which was insufficient for conducting a statistical analysis. We could have relied on clinical examination, which includes assessing aphasia and neglect; however, in the emergency setting, there is a higher likelihood of clinical examination errors. Identifying neglect can be particularly difficult, and distinguishing between aphasia and dysarthria can lead to misinterpretations. Furthermore, determining hemispheric dominance (major/minor) has become increasingly complex since Broca's study in the second half of the 19th century (28), as well as studies by Geschwing and Levitsky (29), Rasmussen and Milner (30), and Ringo et al. (31). This complexity has arisen owing to recent advancements in functional activation MRI, which have revealed a multitude of patterns that challenge the concept of hemispheric dominance (32).

This study has several limitations, including the retrospective nature of the analysis, the small sample due to a monocentric design, the overrepresentation of ASPECT 5 patients in both groups due to the lack of consensus in the indication of MT in lower ASPECT patients during the study period, and the absence of clinical assessments at 3 months to evaluate associative functions in detail (beyond what is possible with the mRS score).

The main strength of this study is that it is the first in the post-thrombectomy era to specifically examine the role of stroke laterality as it relates to the primary endpoint in patients with low-ASPECTS AIS-LVO.

## 5. Conclusion

The clinical outcome in our population of low-ASPECT AIS-LVO strokes was not significantly influenced by stroke laterality. The results of this single-center retrospective study indicate either a lack of strength or equal value in performing mechanical thrombectomy regardless of stroke laterality. A meta-analysis of upcoming RCTs dedicated to low-ASPECT patients might answer the question.

## Data availability statement

The data analyzed in this study is subject to the following licenses/restrictions: Anonymized and coded dataset can be made available upon request. Requests to access these datasets should be directed to victor.dumas@chu-poitiers.fr.

## Author contributions

SB, JN, RG, and SV participated in the proofreading and validation of the article. GH, JP, KS, and WB participated in the data collection. CG conducted the statistical analysis.
